# Unique Characteristics of Patients with Von Hippel–Lindau Disease Defined by Various Diagnostic Criteria

**DOI:** 10.3390/cancers15061657

**Published:** 2023-03-08

**Authors:** Reut Halperin, Liat Arnon, Yehudit Eden-Friedman, Amit Tirosh

**Affiliations:** 1 ENTIRE Endocrine Neoplasia Translational Research Center, Sheba Rd. 2, Ramat Gan 6562601, Israel; 2 Faculty of Medicine, Tel Aviv University, Tel Aviv 6997801, Israel; 3 Division of Endocrinology, Diabetes and Metabolism, Sheba Medical Center, Ramat Gan 5266202, Israel

**Keywords:** pancreatic neuroendocrine tumor, Von Hippel–Lindau disease, hemangioblastoma, renal cell carcinoma, pheochromocytoma, diagnosis

## Abstract

**Simple Summary:**

Von Hippel–Lindau is a rare endocrine (and other organ) multi-neoplasia syndrome. A clinical diagnosis may be defined using different diagnostic criteria. However, the validity of these criteria has not been evaluated thus far. Here, we assess the patient population defined by the main sets of diagnostic criteria and demonstrate the different diagnostic accuracy of each diagnostic criterion based on patients’ characteristics and genetic analysis.

**Abstract:**

Von Hippel–Lindau (VHL) disease diagnosis is based on two criteria sets: International criteria (IC, two hemangioblastomas, one hemangioblastoma plus one visceral lesion, or VHL family history/pathogenic variant plus hemangioblastoma/visceral lesion); or Danish criteria (DC, two clinical manifestations, or VHL family history/pathogenic variant plus hemangioblastoma/visceral lesion). We aimed to compare the characteristics of patients with VHL-related pancreatic neuroendocrine tumor (vPNET) meeting either the clinical Danish criteria only (DOC) or IC to those with sporadic PNET (sPNET). The cohort included 33 patients with VHL (20 vPNETs) and 65 with sPNET. In terms of genetic testing and family history of VHL, 90.0% of the patients with vPNET in the IC group had a germline *VHL* pathogenic variant, and 70.0% had a family history of VHL vs. 20% and 10% in the DOC group, respectively (*p* < 0.05 for both). Patients with vPNET were younger at diagnosis compared with sPNET (51.6 ± 4.1 vs. 62.8 ± 1.5 years, *p* < 0.05). Patients in the IC group were younger at diagnosis with VHL, vPNET, pheochromocytoma, or paraganglioma (PPGL) and renal-cell carcinoma (RCC) than those in the DOC group (*p* < 0.05 for all comparisons). The most prevalent presenting manifestations were hemangioblastoma (42.8%) and PPGL (33.3%) vs. RCC (58.3%) and PNET (41.7%) in the IC vs. DOC groups. In conclusion, patients with vPNET meeting DOC criteria show greater similarity to sPNET. We suggest performing genetic testing, rather than solely using clinical criteria, for establishing the diagnosis of VHL.

## 1. Introduction

Von Hippel–Lindau disease (VHL) is an autosomal dominant inherited disorder caused by a germline pathogenic variant (PV) in the *VHL* gene, a regulator of hypoxia-inducible factors (HIF) [1]. Reduced activity of the VHL protein prevents degradation of HIF, thus promoting cell proliferation and survival, increased glucose utilization, and angiogenesis [2,3]. Clinical manifestations of patients with VHL include hemangioblastomas (HB) of the retina and central nervous system (CNS), renal cell carcinomas (RCC), pancreatic neuroendocrine tumors (PNET), and adrenal pheochromocytomas or paragangliomas (PPGL), as well as cysts of the pancreas, kidney, epididymis, and broad ligament, and endolymphatic sac tumors [4]. 

A clinical diagnosis of VHL can be established based on one of two sets of criteria: the “International” [4,5]; or “Danish” [6] criteria, as detailed in Table 1. The International Criteria are based on Melmon and Rosen’s original criteria published in 1964 [7] requiring any clinical manifestation of VHL, and a CNS HB harbored either by the patient or a family member. The Danish Criteria, developed based on the Danish VHL registry, were revised in 2013 and are in use mainly in Denmark [8]. 

Discovering the genetic background of VHL allowed an extension of these criteria over time [4]. Thus, today both criteria allow diagnosis of VHL if a patient has a genetic or familial background of VHL with at least one clinical manifestation while also allowing a clinically based diagnosis. Yet, the clinical diagnosis differs between the two criteria: while the International clinical criteria require the presence of at least one HB, the Danish criteria allow establishing a clinical diagnosis of VHL based solely on visceral manifestations. 

The leading cause of death in VHL is CNS HB, followed by RCC [9,10,11,12,13], while PNET is an infrequent cause of death. Nevertheless, the presence of metastatic PNET is associated with increased mortality, with tumor size above 3 cm and a pathogenic variant in exon 3 of the *VHL* gene associated with an increased chance of metastatic disease [14,15,16,17,18]. The prevalence of VHL-related PNET (vPNET) is, on average, 5%, reaching up to 17% in specific cohorts [4,5,19,20,21,22]. Compared with sporadic PNET (sPNET), vPNET is diagnosed at a relatively young age, is often non-functional, multifocal, is usually grade 1 or 2, and typically carries a more benign course compared with sPNET [17,23,24,25,26]. Based on the distinct clinical course of vPNET compared to sPNET, supported by retrospective data on differences in mortality risk [13], specific management recommendations for vPNET were developed by Laks et al. in 2022 [27], which differ from the guidelines for sPNET management in various parameters [28,29]. 

In the current study, we aimed to compare the characteristics of vPNET, as defined by the clinical definitions of International and Danish Criteria for the diagnosis of VHL. We compared both subgroups to sPNET to assess whether the more liberal definition of VHL, based on the Danish criteria, defines a clinically distinct patient population. 

## 2. Materials and Methods

This research was approved by the Sheba MC Institutional Review Board (SMC-18-5735 and SMC-18-5674). Patients, who required genetic testing as part of this study, signed a written informed consent. This was a retrospective study, based on data retrieved using the MD Clone system (Beer-Sheva, Israel), of patients diagnosed with PNET between 1995 to 2021 at a tertiary medical center. Data collected included demographic characteristics (age at diagnosis, sex, and diagnoses of VHL manifestations); PNET-specific characteristics: location of the tumor within the pancreas; grade (differentiation, percentage of positive staining for KI-67, and mitotic index); and stage (based on the 8th Edition of the American Joint Committee on Cancer [30]); imaging results: computed tomography (CT) and/or magnetic resonant imaging (MRI) scans; ultrasonography (US); and/or positron emitting tomography (PET). The parameters collected for each modality included timing and report of progression, regression, or stability based on expert radiologist interpretation; intervention types: none; pharmacological; radionuclide; invasive radiology or surgical; and mortality of any cause. In addition, data on VHL family history and results of *VHL* germline genetic testing (indels or point pathogenic variants) were gathered.

The patients were sub-grouped based on whether they harbored vPNET or sPNET and whether the VHL diagnosis was determined by the Danish criteria (DC) or the International criteria (IC, see Table 1 for definitions of each criterion). Due to an overlap between the IC and DC, all patients diagnosed according to the IC also fulfill the DC. We, therefore, formed the following comparison groups: International (IC, patients fulfilling the international clinical criteria); Danish (DC, all patients fulfilling the Danish clinical criteria); Danish-only (DOC, patients fulfilling the Danish clinical criteria but not the International clinical criteria); and sPNET (fulfilling neither criteria for VHL diagnosis). Progression-free survival was calculated as the interval (in months) between the date of diagnosis and the timing of the earliest report of progression based on any imaging modality.

There were nineteen patients in the sPNET group with partial or missing pathology data. Six patients did not undergo biopsy: four patients had poor health status, and two had a longstanding stable small PNET, in which the diagnosis was based on the pathognomonic radiological appearance. Three patients had indeterminate grades: two had a well-differentiated PNET per pathology report, not defining KI67 or mitotic index, and one patient underwent endoscopic ultrasound-guided biopsy, but the material was too scarce to allow grading. For ten patients, grading was not defined, as they were sampled before 2010 when the World Health Organization defined the NET grading system. 

Eight patients with vPNET did not undergo biopsy/surgical resection of the lesion, including seven from the IC group. The vPNET in these patients was not sampled following institutional multidisciplinary team discussion, considering the diagnosis of VHL, the high likelihood for vPNET, and the tendency for diagnosing vPNETs using non-interventional manners. Two patients with no grades available were from the DOC group: one patient, 81 years-old male, was at high risk for intervention, and for the other, tumor tissue quantity was not sufficient for determining tumor grade. In two additional patients from the DC group, pathological data were not available. 

### Statistical Analysis

The statistical analysis was performed via SPSS Statistics (version 20.0.0, IBM, 2011, Armonk, NY, USA). Continuous variables with normal distribution were compared via the Student’s *t*-test, and categorical parameters were compared using the chi-square test or Fisher’s exact test. Continuous variables are presented as mean ± standard error of the mean (SEM) unless stated otherwise, and categorical parameters are presented as n (%). Non-parametric tests were used as appropriate. Two-tailed *p*-value < 0.05 was set as a threshold for statistical significance.

## 3. Results

### 3.1. sPNET vs. vPNET

Patients and tumor characteristics of both sPNET and vPNET are presented in Table 2. Thirty-three patients with a diagnosis of VHL according to either the International (n = 21) or Danish-only (n = 12) criteria were identified, of which 20 patients were diagnosed with PNET (mean age at diagnosis with vPNET was 51.6 ± 4.1 years, 60.0% males). In 65 patients with sPNET (56.9% males) mean age at diagnosis with PNET was 62.8 ± 1.5 years. The vPNETs were detected at a younger age compared with sPNET (*p* < 0.001). There was a significant difference in the stage but not in the grade between sPNET and vPNET (Table 2). A higher fraction of patients with sporadic PNET had stage III/IV diseases as compared with patients with vPNET (*p* = 0.02). There was a smaller proportion of vPNET located at the body/tail of the pancreas vs. the sPNET group (36.8% vs. 64.6%, *p* = 0.012). 

### 3.2. DOC vs. IC Criteria–PNET Characteristics

To assess the differences between patients diagnosed with VHL based on the DOC vs. IC, we compared their demographic characteristics (Table 3) and the various manifestations of VHL (Table 4) between the two groups. Patients with IC-based diagnosis were younger at diagnosis with PNET compared with the DOC group (40.5 ± 5.5 vs. 62.6 ± 4.1 years, *p* = 0.003, respectively). Stage, grade, and PNET location did not differ between groups (Table 3). 

### 3.3. VHL Characteristics in Patients with PNET-DOC vs. IC Criteria

Nine (90%) of the ten patients that underwent germline DNA genotyping of the VHL gene in the IC group had a pathogenic variant, as compared to one patient out of five tested (20%) in the DOC group (*p* = 0.007). Among those genetically diagnosed with VHL, 15 had a missense PV, and three had a deletion (additional two patients had an unknown PV type). The most frequent pathogenic variant identified was *p*. Gly93Arg (identified in five patients of two kindreds). Seventy percent (7/10) of the patients diagnosed with VHL based on the IC had a family history of VHL manifestations in a total of 15 different kindreds, compared with ten percent (one patient) in the DOC group (*p* = 0.006, Table 4). Age at diagnosis of VHL was younger among patients diagnosed based on the IC vs. DOC (26.6 ± 4.1 vs. 58.8 ± 4.3 years, *p* < 0.001, respectively). Furthermore, diagnoses with RCC and PPGL were also made at a significantly younger age in the IC compared with the DOC group (*p* = 0.05 for RCC and *p* = 0.01 for PPGL, Table 4). By definition, all patients (10/10) in the IC group had CNS HB, and 30.0% (3/10) had retinal HB compared with none in the DOC group. 

### 3.4. Clinical Characteristics of Patients Diagnosed with VHL Based on the DOC and IC

Out of the 33 patients diagnosed with VHL, 21 were diagnosed according to the IC and 12 according to the DOC (Table 5). Relevant family history was reported in 61.9% of the patients in the IC group compared with one patient (8.3%) in the DOC group (*p* = 0.003). Of those tested, a confirmed pathogenic VHL variant was found in 95.3% percent of patients in the IC group vs. 20.0% in the DOC group (*p* < 0.001). Age at diagnosis of VHL was significantly younger in the IC (22.6 ± 2.3 years) vs. DOC group (57.1 ± 4.0 years, *p* < 0.001). This was also true for age at diagnosis with PNET (*p* = 0.003), RCC (*p* = 0.009), and PPGL (*p* < 0.001, Table 5). The most frequent first VHL manifestation in the IC group was CNS HB (42.8%), followed by PPGL (33.3%) vs. RCC (58.3%) and PNET (41.7%) in DOC. 

## 4. Discussion

In this study, we aimed to assess the difference in the population selected by using different sets of VHL clinical diagnostic criteria. We assessed the difference in terms of patient characteristics and PNET characteristics, both between the two criteria sets (Danish and International) and vs. sporadic PNET.

We show that the patients diagnosed solely based on the DOC are mostly VHL-mutation negative (albeit the small number of patients undergoing germline genetic evaluation), compared with ~95% positivity among those diagnosed based on the IC. In general, VHL-associated PNETs were diagnosed at a younger age than sporadic PNETs. However, we found that this is criteria-dependent, as PNETs were detected at a younger age in patients with IC-VHL vs. DOC-VHL, while age at DOC-VHL PNET diagnosis was comparable to sPNET diagnosis. Similarly, the age at diagnosis of VHL, PNET, RCC, and PPGL was significantly younger for patients in the IC-VHL vs. DOC-VHL groups.

The finding that PNET arises at a younger age in VHL patients compared with patients with the sporadic disease is well documented [24,25], with a mean age of vPNET at the presentation of 31–49 years [10,17,20] and of sPNET of 60.2 ± 13 years [15]. Hence, the absence of such differences between DOC-vPNET and sPNET raises a question on the validity of these clinical criteria. In contrast to the distinctly younger age of vPNET diagnosis vs. sPNET, the difference in pancreatic tumor location was not previously recognized. In a retrospective study based on Surveillance, Epidemiology, and End Results (SEER) database, approximately two-thirds of PNETs were located at the body or tail of the pancreas, with worse prognosis in those located at the head of the pancreas, possibly explained by the greater mass of Langerhans islets in the pancreatic body and tail compared with the head of pancreas [31]. Since the composition of the endocrine cells within the pancreatic islets differs between pancreatic anatomic locations [32], the higher rate of pancreatic head PNET in the context of VHL may suggest a specific cell of origin that is more abundant in the pancreatic head islets.

In the current study, we found striking differences in VHL characteristics between patients diagnosed according to the IC and DOC criteria. The IC group had a significantly younger age of diagnosis of VHL, PNET, PPGL, and RCC. An earlier VHL diagnosis may be attributed to the young age of occurrence of CNS HB (averaging around 30 years of age [9,20]) and retinal HB (peaking before 20 years of age [8]), that were not included in the DOC group by definition. 

PNET is a rare diagnosis, 0.8 per 100,000 patients [33], with increasing incidence in the past decades, mostly of low-grade PNET with a decrease in high-grade PNET seen over time [34]. This rise is at least partly related to trends in imaging and improved recognition in histology [35]. RCC is a common diagnosis accounting for 2% of all cancer diagnoses in the general population, with a rising incidence estimated to be 9.6–10.9 per 100,000 patients, with most cases discovered incidentally on imaging [36]. Overall, from all reported cases, only <5% of all RCC cases and <1% of all PNET cases are VHL-related [8]. In the DOC group, 80% of patients had RCC and PNET, both at much higher rates than that reported in patients with genetically proven VHL [4]. The high prevalence and the difference in age of diagnosis of RCC and PNET between these VHL groups may stem from the high frequency of RCC and rising frequency of PNET in the general population, leading to an erroneous clinically-based diagnosis of VHL of patients that, in fact, co-carry sporadic RCC and PNET. Indeed, when looking at the genetic basis of VHL diagnosis, we found that while 95% of the patients in the IC group that underwent genetic testing carried a pathogenic variant, only 20% (one patient) of those tested in the DOC group carried a variant, this was also true for family history of VHL. 

It should be noted, though, that patients in the DOC group were less likely to undergo genetic testing. Clinically, patients diagnosed with VHL according to the Danish criteria were older with incidental identification of RCC and PNET; thus, they were rarely suspected of having the genetic syndrome. While this is reflected in the low likelihood of having a *VHL* gene pathogenic variant, it also explains the low rate of genetic testing performed in this patient population. We, thus, suggest refraining from diagnosing patients with VHL based on the combination of RCC and PNET without confirming the diagnosis with the identification of a pathogenic variant. VHL testing genotyping is a highly accurate test for ruling out a diagnosis of VHL. DNA sequencing (Sanger) identifies 89% of the variants, with only 11% potential false negatives with this technique due to whole exon or whole gene deletions [37]. Analyses for copy number alterations might be needed in cases with high clinical suspicion, with negative results per Sanger. The rare possibility of mosaicism should also be considered, necessitating the use of next-generation sequencing [38].

Further supporting this suggestion is the revision of VHL diagnosis in the current Danish VHL guidelines (published in 2022), now allowing a clinical diagnosis based only on a combination of HB and another clinical manifestation [39]. In our view, in the rare cases in which genetic testing is not feasible and VHL diagnosis is based solely on clinical criteria, VHL diagnosis should be supported by implementing a maximal age cutoff. For example, VHL penetrance is over 95% by age 60 years and 99% by age 70 years [9]; therefore, a sole clinical-based VHL diagnosis in a patient over 70 years old should be avoided. 

This study has several limitations. First, this is a retrospective study, and as such, not all patients had a complete medical record, specifically regarding the grade of vPNET. The lack of data on PNET grade for the majority of patients with vPNETs stems from the unique diagnostic approach for PNET in patients with VHL as delineated in the vPNET management guidelines [27] explains the high rate of non-invasive diagnosis of vPNET in the IC group, in which 7/10 patients did not undergo biopsy of their pancreatic lesion, and the diagnosis of PNET was based on the pathognomonic appearance on imaging as a biopsy in not regularly recommended for patients with VHL. Second, while this is the practice these days, we are unable to carry genetic testing to all patients diagnosed with VHL in retrospect. Third, the VHL patient population in this study may be characteristic of our clinic and differ from other geographic sites since the genotype distribution is population- and geography dependent, albeit the large number of kindreds reported in this study may suggest differently. Fourth, patients with VHL undergo regular screening for vPNET and, thus, detection is expected at an earlier stage (and perhaps also age) compared to patients with sPNET. Finally, the sporadic group had a relatively low frequency of high-grade PNET, most probably stemming from documentation bias due to the referral of high-grade patients for follow-up at the oncology institute in previous years. 

## 5. Conclusions

Diagnosis of VHL according to different criteria results in different patient characteristics and may include patients with sporadic disease. Our findings question the role of clinical diagnosis of monogenic hereditary syndromes with a known causing gene. Such diagnosis is straightforward and highly affordable and, thus, should be performed considering its huge impact on patient management. The follow-up required for patients with VHL is lifelong and comprises numerous tests and clinic visits, associated with the heavy emotional and administrative burden on the patients and a profound impact on their lives and their families’ daily living. In addition to this burden, the diagnosis of VHL affects the decision-making of its various related manifestation. This includes, for example, less stringent sampling of new lesions, a different threshold for surgical interventions, and VHL-targeted medical therapy. Hence, the diagnosis of VHL should be determined using the most specific clinical and genetic assessment tools with the highest positive predictive value. Based on our data, the clinical Danish criteria specificity is not comparable to genetic testing and is inferior to the International criteria that are based on unique clinical manifestations of VHL (mainly multiple hemangioblastomas). Thus, we suggest using genetic testing for the diagnosis of VHL, and in cases in which such resources are not available—using the international clinical criteria. 

## Figures and Tables

**Table 1:** Clinical criteria for the diagnosis of Von Hippel–Lindau disease based on the International and Danish VHL diagnostic criteria.

International Criteria	Danish Criteria
Multiple CNS or retinal hemangioblastomas or CNS or retinal HB and one visceral lesion ^a^ or Positive family history or a pathogenic variant in the *VHL* gene and one CNS or retinal HB or visceral lesion	Any two VHL-related manifestations ^b^ or Pathogenic variant in the *VHL* gene and any clinical manifestation or At least one first-degree relative with VHL and any one clinical manifestation

^a^ Clear-cell renal cell carcinoma, pheochromocytoma or paraganglioma, or pancreatic neuroendocrine tumor. ^b^ Central nervous system (CNS) hemangioblastoma (HB), retinal hemangioblastoma, endolymphatic sac tumor, renal cell carcinoma, pheochromocytoma, paraganglioma or glomus tumor, pancreatic neuroendocrine neoplasms, and/or multiple cysts.

**Table 2:** Demographic and clinical comparison between VHL-related and sporadic PNET.

	Sporadic	VHL	*p* Value
n	65	20	
Male sex (%)	37 (56.9)	12 (60.0)	0.81
Age at diagnosis of PNET (years)	62.8 ± 1.5	51.6 ± 4.1	0.002
Grade G1 + G2/G3	37 (80.4)/9 (19.6) ^a^	10 (100.0)/0 (0.0) ^b^	0.72
Stage I-II/III-IV	35 (54.7)/29 (45.3)	16 (84.2)/3 (15.7)	0.02
PNET location head /body + tail	20 (30.8)/42 (64.6)	12 (63.1)/7 (36.8)	0.012
Surgery performed	33 (53.9)	6 (30.0)	0.1
Pharmacological treatment	31 (50.8)	2 (10.0)	0.002
PNET advancement	25 (43.8)	5 (25.0)	0.27
Progression-free survival (months)	40.3 ± 8.5	30.1 ± 7.5	0.36

Continuous variables are reported as mean ± standard error of mean (SEM), and categorical variables are reported as n (%). Percent was calculated out of the available data. ^a^ Six patients did not undergo biopsy/surgical resection, three had insufficient data on pathology reports to allow grading, and seven were operated on before 2010 when the WHO defined the NET grading system. ^b^ Eight patients with vPNET did not undergo biopsy/surgical resection of the lesion, and the additional two had no grade available (high risk for biopsy and insufficient tissue for defining grade). PNET = pancreatic neuroendocrine tumor.

**Table 3:** VHL-related PNET characteristics–comparison between IC, DC, and DOC.

	IC	DOC	DC	*p* Value *
n	10	10	20	
Males	6 (60.0)	6 (60.0)	12 (60.0)	0.81
Age at PNET diagnosis (years)	40.5 ± 5.5	62.6 ± 4.1	51.6 ± 4.1	0.003
PNET as 1st manifestation	1 (10.0)	5 (50.0)	6 (30.0)	0.051
Grade G1/G2 *	1 (33.3)/2 (66.7)	3 (50.0)/3 (50.0)	4 (44.4)/5 (55.5)	NS
Stage I/II vs. III/IV	7 (77.8)/2 (22.2)	9 (90.0)/1 (10.0)	16 (84.2)/3 (15.7)	0.46
PNET location head/body + tail	7 (70.0)/3 (30.0)	5(55.5)/4 (44.4)	12 (63.1)/7 (36.8)	0.51
Surgery performed	3 (30.0)	3 (37.5)	6 (30.0)	NS
Pharmacological treatment	2 (20.0)	0 (0.0)	2 (10.0)	NS
PNET advancement	2 (20.0)	3 (30.0)	5 (25.0)	0.61

Continuous variables are reported as mean ± standard error of mean (SEM), and categorical variables are reported as n (%). Percentages were calculated from the available data. PNET = pancreatic neuroendocrine tumor. Subgroups: International (IC); Danish (DC); and Danish-only criteria (DOC). * Statistical comparison between IC and DOC groups.

**Table 4:** Comparison of VHL manifestations in patients with a concurrent diagnosis of PNET based on the IC vs. DOC.

		IC	DOC	DC	*p* Value *
VHL	N	10	10	20	
	Age (years)	26.6 ± 4.1	58.8 ± 4.3	42.7 ± 4.7	<0.001
	Known family history	7 (70.0)	1 (10.0)	8 (40.0)	0.006
	Confirmed VHL PV	9/10 (90.0) ^#^	1/5 (20.0)	10/15 (66.7)	0.007
RCC	N	6 (60.0)	8 (80.0)	14 (70.0)	0.32
	Age (years)	35.7 ± 6.4	58.9 ± 4.8	48.1 ± 4.7	0.005
	Multiple/met	4 (66.7)	1 (12.5)	5 (35.7)	0.36
	Surgical treatment	3 (50.0)	5 (62.5)	8 (57.1)	0.64
	First manifestation	0 (0.0)	6 (60.0)	6 (30.3)	0.01
CNS HB	n	10 (100.0)	NA	10 (50.0)	
	Age (years)	30.7 ± 4.3	NA	30.7 ± 4.3	
	Multiple	6 (60.0)	NA	6 (60.0)	
	Surgical treatment	9 (90.0)	NA	9 (90.0)	
	First manifestation	7 (70.0)	NA	7 (70.0)	
Optic HB	N	3 (30.0)	NA	3 (15.0)	
	Age (years)	27.7 ± 10.1	NA	27.7 ± 10.1	
	Multiple	2 (66.7)	NA	2 (66.7)	
	Surgical/laser treatment	3 (100.0)	NA	3 (100.0)	
	First manifestation	0 (0.0)	NA	0 (0.0)	
PPGL	n	3 (30.0)	2 (20.0)	5 (25.0)	0.61
	Age (years)	22.1 ± 6.8	67.2 ± 8.1	40.2 ± 11.6	0.01
	Bilateral	2 (66.7)	0 (0.0)	2 (40.0)	0.71
	Surgical treatment	3 (100.0)	2 (100.0)	5 (100.0)	0.54
	First manifestation	2 (20.0)	1 (10.0)	3 (15.0)	0.53

Continuous variables are reported as mean ± standard error of mean (SEM), and categorical variables are reported as n (%). Percentages were calculated from the available data. CNS = central neural system. HB = hemangioblastoma. PNET = pancreatic neuroendocrine tumor. PPGL = paraganglioma or pheochromocytoma. RCC = renal cell carcinoma. VHL = Von Hippel–Lindau disease. International (IC); Danish (DC); and Danish-only criteria (DOC). ^#^ One patient underwent genetic testing, but results could not be confirmed. Subgroups: International (IC); Danish (DC); and Danish-only criteria (DOC). * Statistical comparison between IC and DOC groups.

**Table 5:** Comparison of VHL manifestations between patients diagnosed with VHL based on the IC or the DOC.

		IC	DOC	DC	*p* Value *
VHL	n	21	12	33	
	Male	11 (52.4)	8 (66.7)	19 (57.6)	0.42
	Age years	22.6 ± 2.3	57.1 ± 4.0	34.5 ± 3.6	<0.001
	Known family history	13 (61.9)	1 (8.3)	14 (42.4)	0.003
	Confirmed VHL PV	20/21 (95.3)	1/5 (20.0)	21/26 (80.8)	<0.001
PNET	n	10 (47.6)	10 (83.3)	20	0.043
	Age years	40.5 ± 5.5	62.6 ± 4.1	51.6 ± 4.2	0.003
	Multiple/met	0 (0.0)	2 (20.0)	2 (10.0)	NS
	Surgical treatment	3 (30.0)	3 (30.0)	6 (33.3)	0.26
	First manifestation	1 (4.8)	5 (41.7)	6 (18.1)	0.008
RCC	n	9 (42.8)	10 (83.3)	19 (57.6)	0.023
	Age years	35.6 ± 6.3	56.8 ± 4.5	45.6 ± 4.5	0.009
	Multiple/met	7 (77.8)	1 (10.0)	8 (42.1)	0.003
	Surgical treatment	6 (66.7)	7 (70.0)	13 (68.4)	0.87
	First manifestation	0 (0.0)	7 (58.3)	7 (21.1)	<0.001
CNS HB	n	21 (100.0)	NA	21 (63.6)	
	Age (years)	29.3 ± 2.5	NA	29.3 ± 2.5	
	Multiple	10 (47.6)	NA	10 (47.6)	
	Surgical treatment	13 (61.9)	NA	13 (61.9)	
	First manifestation	9 (42.8)	NA	9 (27.3)	
Retinal HB	n	11 (52.8)	NA	11 (33.3)	
	Age (years)	23.4 ± 3.1	NA	23.4 ± 3.1	
	Multiple	6 (54.5)	NA	6 (54.5)	
	Surgery\laser	10 (90.9)	NA	10 (90.9)	
	First manifestation	4 (19.0)	NA	4 (12.1)	
PPGL	n	9 (42.8)	4 (33.3)	13 (39.4)	0.59
	Age (years)	22.2 ± 2.8	60.2 ± 5.4	33.9 ± 5.75	<0.001
	Bilateral	7 (77.8)	0 (0.0)	7 (53.8)	0.021
	Surgical treatment	9 (100.0)	4 (100.0)	13 (100.0)	1
	First manifestation	7 (33.3)	1 (8.3)	8 (24.2)	0.11

Continuous variables are reported as mean ± standard error of mean (SEM), and categorical variables are reported as n (%). Percentages were calculated out of the available data. CNS = central neural system. HB = hemangioblastoma. PNET = pancreatic neuroendocrine tumor. PV = pathogenic variant. PPGL = paraganglioma or pheochromocytoma. RCC = renal cell carcinoma. SEM = standard error of mean. VHL = Von Hippel–Lindau disease. Subgroups: International (IC); Danish (DC); and Danish-only criteria (DOC). * Statistical comparison between IC and DOC groups.
